# Improving person-centered care for people with multimorbidity: the potential of participatory learning and action research

**DOI:** 10.1177/26335565231207075

**Published:** 2023-10-25

**Authors:** Anne MacFarlane, Marianne McCallum, Moira Stewart

**Affiliations:** 1 Public and Patient Involvement Research Unit and WHO Collaborating Centre for Participatory Health Research with Refugees and Migrants, School of Medicine, 8808 University of Limerick, Limerick, Ireland; 2 Department of General Practice and Primary Care, 3526 University of Glasgow, Glasgow, Scotland; 3 Department of Family Medicine, Centre for Studies in Family Medicine, Western University, London, ON, Canada

**Keywords:** Multi-morbidity, person-centered care, patient involvement, participatory health research

Multimorbidity and the pressure it applies to health systems is one of the most significant challenges facing global health systems. Crucially multimorbidity is not experienced equally across populations, being more prevalent in areas of high socioeconomic deprivation, where it begins at a younger age.^1^ In addition, communities experiencing high socio-economic deprivation have higher rates of mental health co-morbidities which impacts healthcare utilisation, healthcare experiences and outcomes.^1^

Person centered care is of central importance for addressing the needs of people who experience multimorbidity, especially in populations experience high socio-economic disadvantage. Yet, this is not being achieved for multiple reasons including conceptual limitations in the field.^2^ Despite a rich literature, there is *conceptual ambiguity* about person centered care with different terms in use in different places at different times. To address this, recent reviews^3,4^ have critically examined conceptual frameworks over 20 years to explore shared and differential features. Both reviews concluded that, in fact, the diverse terminology is underpinned by remarkably similar themes. These include the central importance of engaging with patients about their thoughts and feelings about health and healthcare; their family and social context; their goals and preferences for management and treatment; and their experiences of therapeutic relationships including attention to emotions and power-sharing. Further, the reviews elucidated the importance of considering the clinician as person and the co-ordination of care.

There is also *conceptual partiality* in the field of person centered care because patients have not had a voice in its conceptualisation. Of all 159 papers reviewed by Sturgiss et al.,^4^ only 15% (25 papers) explicitly included the patient perspective in their analysis/concept creation. This “lack of the perspective of the patient… appears to be in direct conflict with the stated intentions of those interested in increasing (patient) centredness.”^4^ This finding reveals that professionals have dominated conceptualisation and points to the need for more participation of patients in the co-creation of definitions about their care.

Opportunities for co-creation are particularly important for patients who experience multimorbidity from areas with high socio-economic deprivation because wider social experiences shape health: society’s dominant groups (white, middle-class, cis-gender, housed people) are prioritised and normalised in health policy, service design and delivery. This means that ‘ways of being’ within more socially vulnerable communities are dismissed, or even considered inappropriate and deviant. This creates recursive negative, experiences at the point of healthcare access and delivery diminishing the empathy of healthcare providers and undermining the organisation and delivery of co-ordinated care, all of which exacerbates health inequities.^5^ Thus, if we are serious about improving implementation of person centered care for people with multimorbidity who live in areas of high socio-economic deprivation, we must integrate their voices into the co-creation of person centered concepts and practice.

There are strong policy imperatives for patient voice in health including public and patient involvement/patient engagement in research and shared decision-making. Yet, here too, there is an implementation problem: these are not routine, normalised ways of working in academic and clinical settings.^6^ This means that new directions are needed to improve the implementation of patients’ voices in order to improve the implementation of person centered care. This is not an insignificant challenge. However, we cannot let it paralyse us. Instead, we can let it tantalize us to consider new approaches for meaningful participation of people who experience multimorbidity in these co-creation activities.

Participatory health research is a social justice research paradigm that aims to bridge the gap between knowledge and action. Participatory health research advocates that those whose lives and work are affected by an issue collaborate together to generate local, contextually relevant evidence for action.^7^ Participatory health research centralises the involvement of local experts with lived experience of the issue of interest (“insiders”) alongside those who are more usually regarded as experts: people from statutory agencies or other settings (“outsiders”). It is an umbrella term for a broad and expanding ‘family’ of approaches that share core values of inclusivity; that pay active attention to power asymmetries between insiders and outsiders; and that aim to reverse dynamics that underscore ‘helicopter’ research i.e., extractive research based on outsiders’ priorities and needs, which do not serve patient and community groups well. For meaningful partnership, insiders should have a voice in setting the research agenda and need to be involved from start to finish so that their expertise and opportunity to share decisions is incorporated throughout the project.^7^ Within the family of participatory health research, there are rich resources for designing partnerships from project start to finish. Here, we draw attention to the resources provided by one approach, Participatory Learning and Action (PLA) research. PLA has been used successfully in primary care studies to involve refugees and migrants in research and serves as a concrete example for multi-morbidity researchers who wish to consider participatory approaches in their work.

PLA is a practical, adaptive, action-oriented research approach.^8^ It focuses on bringing diverse groups and individuals together in a safe space to focus on an issue of joint concern so that they can learn, work and act together in a collaborative and democratic manner. PLA seeks to flatten hierarchies between such groups and individuals through (i) a participatory mode of engagement that concentrates on building trust and relationships and (ii) the use of participatory tools and techniques to structure respectful dialogues. These techniques involve the co-creation of visual charts with post-its and visual images to summarise key information and emotions. This process supports brainstorming, on-the-spot ‘co-analysis’ of emerging ideas and themes and democratic ranking of options.^8^

In the field of refugee and migrant health research, PLA has been used in the USA and Europe to explore research priorities for primary care clinical networks leading to the identification of unanticipated community priorities.^9^ PLA has been used in Steering Group meetings to build relationship between its members and inform the collaborative selection of case study sites for qualitative fieldwork and, then, been used by trained community researchers to support data generation and co-analysis at those sites with communities who experience high socio-economic disadvantage.^10^ PLA has been used to inform the selection, adaptation and introduction of guidelines in primary care clinical settings that led to concrete and sustained improvements in cross-cultural communication in those practices.^8^

Evaluations of PLA as a process^7,8^ indicate that the PLA enriches relationships between insiders and outsiders and can lead to anticipated benefits, such as changes in practices and ripple effects for individuals (e.g., greater confidence) and groups (e.g, new networks to support knowledge translation). One critique of PLA is that it takes time. This is true because of its emphasis on relationships and the centrality of dialogues for learning, which take time. This can be challenging given the lack of resources for clinicians and others from health sector organisations to spend adequate time with patient and community members for dialogues about their shared concerns.^6^ However, given the pressing challenges of multimorbidity and widening inequities, we can consider an alternative view: PLA may be a tremendous *investment of time* Thus, we propose that it warrants further investigation as a research approach to create new spaces for participation in the field of multimorbidity research: Is PLA equally effective at different stages of the research cycle? How does it compare to other participatory approaches? What resources are required to implement it as a research approach in the field of multi-morbidity?.

We see the value in exploring these questions because PLA has potential for addressing conceptual ambiguity and conceptual partiality in multimorbidity research given the resources it offers for creating radical spaces for dialogues with people who experience multimorbidity and who live in areas with high socio-economic disadvantage. These dialogues can elicit deep insight into the existing (and emergent) themes for person centered care: their thoughts and feelings, family and social contexts, their goals and preferences for management and treatment; and their experiences of therapeutic relationships including attention to emotions and power-sharing. Further, PLA can be used to create spaces that also acknowledge the clinician as person and enable them to share their perspectives on the organisation and delivery of care in pressurised clinical environments and health systems that do not always support co-ordination of care. In this way, PLA has potential to elicit a new collation of actors and a collage of expertise from different perspectives leading to more comprehensive, actionable knowledge to strengthen the concept and practice of person centered care for all.
